# Starting 2012: Sod2, DGAT1, p53, SPARC, QTL and reprogramming in aging

**DOI:** 10.18632/aging.100429

**Published:** 2012-01-01

The 2012 year has already witnessed several scientific breakthroughs and discoveries. Diabetes, caused by destruction of insulin-producing beta-cells, may require life long injections of insulin and even that does not preclude diabetic complications. Transplantation of beta-cells is one of the options but transplant rejection is a problem. Could we collect skin cells from a diabetic patient, induce them to become stem cells and then induce these stem cells to become beta-cells. Given that diabetic cells suffer from metabolic abnormalities, this may seem to be impossible, especially in elderly patients with type II diabetes. But this is exactly what was reported in this issue of *Aging*. In elderly type 2 diabetes patients, reprogramming of keratinocytes ensures a senescence-privileged status yielding stem cells proficient for regenerative applications (see Ohmine et al, Aging 2012; 4: this issue). Type II diabetic patients suffer from insulin-resistance. Insulin resistance may be caused by feedback inhibition of signals by over-activated mTOR. And mTOR is involved in aging. Not surprisingly, cells from old individuals can be signal-resistant. As reported in this issue of *Aging* by Nakamura et al, age-related resistance of skeletal muscle-derived progenitor cells to SPARC (an autocrine differentiation factor) may explain a shift from myogenesis to adipogenesis (Aging 2012; 4: this issue). This in turn may explain age-related sarcopenia. Interestingly, inhibition of mTOR may decrease sarcopenia (Aging 201; 3:83-84.) probably due to restoration of signal-sensitivity. The challenge is to restore signal-sensitivity pharmacologically, using pulse-treatment with rapamycin as suggested previously (Rejuvenation Res. 2008;11:801-808). Alternatively, calorie restriction may also restore the responsiveness to signals and extend lifespan. As reported in this issue of *Aging* by Streeper et al, the deficiency of the triglyceride synthesis enzyme acyl CoA:diacylglycerol acyltransferase 1 (DGAT1), which promotes leanness, also extends longevity without limiting food intake. Such mice are spared from age-related obesity, elevated IGF-1 and inflammation in white adipose tissue. This was accompanied by increased mean and maximal life spans of 25% and 10%, respectively (Aging 2012; 4: this issue). It was recently shown that CR decreased a number of senescent cells in the organism (Aging 2010; 2:555-566). In this issue of *Aging*, Campisi and co-workers demonstrated that mitochondrial oxidative stress caused by Sod2 deficiency promotes cellular senescence and aging phenotypes in the skin (Velarde et al. Aging 2012; 4: this issue). This excellent model may be helpful to investigate whether CR might blunt the effect. As reported by Altilia et al in this issue of *Aging*, cells bearing p53R72 accumulate lower amount of mtDNA damage. This paper suggests that the polymorphism of p53 at codon 72 affects the accumulation of mtDNA mutations and may contribute to in vivo accumulation of mtDNA mutations (Aging 2012; 4: this issue). Finally, age-related macular degeneration (AMD) and cataract are common age-related diseases in humans. In this issue of *Aging* Korbolina et al reported quantitative trait loci (QTLs), which affect early-onset cataract and retinopathy in senescence-accelerated OXYS rats, comparable to human AMD and senile cataract. The 2012 year is just started. We expect to see more discoveries in forthcoming months.

